# Performance of the Paediatric Trauma Score on survival prediction of injured children at a major trauma centre: A retrospective Colombian cohort, 2011–2019

**DOI:** 10.1016/j.lana.2022.100312

**Published:** 2022-06-29

**Authors:** Ana De los Ríos-Pérez, Alberto García, Laura Cuello, Sara Rebolledo, Andrés Fandiño-Losada

**Affiliations:** a Program in Methodology of Biomedical Research and Public Health, Universitat Autònoma de Barcelona, Barcelona, Spain; b Fundación Valle del Lili University Hospital, Cali, Colombia; c Faculty of Health Sciences, Universidad Icesi, Cali, Colombia; d Faculty of Health, Universidad del Valle, Cali, Colombia; e Cisalva Institute, Faculty of Health, Universidad del Valle, Cali, Colombia

**Keywords:** Injuries, Paediatric trauma, Trauma severity indices, Trauma score, Pediatric trauma score, Mortality, Survival prediction

## Abstract

**Background:**

Despite improvements in children's health due to a reduction in infections, trauma continues to cause many deaths among adolescents. Strategies to mitigate morbidity and mortality from trauma include severity scores to classify and refer patients to the appropriate hospitals to provide better management; however, these strategies have not been assessed in Colombian children. This study aimed to describe the characteristics and outcomes of injured children and evaluate the performance of the Pediatric Trauma Score (PTS) in predicting survival at a major trauma centre in a Colombian city.

**Methods:**

This was a retrospective cohort study of children aged <18 years who were treated for injuries at a hospital in Colombia. The primary outcome was 30-day mortality. A simple logistic regression model was used with PTS as the predictor variable and vital status at discharge as the outcome variable. PTS performance was assessed by discrimination using the area under the receiver-operating characteristic (AUROC) curve and by calibration using the Hosmer-Lemeshow (HL) goodness-of-fit test.

**Findings:**

A total of 1047 children were admitted. The median age was 12 years (interquartile range [IQR]=5–15); 73·7% were male, and 66·1% had blunt trauma. The most frequent cause of injury was traffic accident (31·5%) followed by assaults (29%). Mortality was 5·9%; 61·3% of these deaths occurred in adolescents between 15 and 17 years of age and 71% of deaths in this age group were due to injuries from a firearm. The PTS had a median of 7 (IQR=5–9), an AUROC of 0·93, and good calibration (HL=7·97, *p* = 0·33).

**Interpretation:**

The highest proportion of trauma and death occurred among adolescents. Interpersonal violence was the most frequent cause of death in this age group. The PTS showed good predictive power for survival, with excellent discrimination and good calibration.

**Funding:**

None.

**Research in context**

**Evidence before this study**

This study was conducted in 2019. Before its initiation, a PubMed search was conducted using the search terms ("Pediatrics"[Mesh] OR pediatric* OR child*) AND (trauma OR injur*) AND ("survival prediction") without language or year of publication restrictions. No study has assessed the prediction of survival in injured children or the performance of PTS in their care in Latin America. Few studies on paediatric trauma were found from Colombia; most descriptive and used a combination of adult and paediatric subjects, and none applied trauma scores in children. We repeated the search in July 2021, and again found no publications in Latin America.

**Added value of this study**

Research on paediatric trauma in Colombia is scarce, limiting the availability of data to enable the proposal of intervention strategies that mitigate the incidence of trauma and its devastating consequences. This study contributes to scientific evidence in Colombia and Latin America and is one of the region's largest cohorts of paediatric trauma studies. Unlike most studies reported worldwide, this study showed a significant degree of firearm violence among adolescents; firearm violence was the leading cause of death. Therefore, firearm violence is a relevant public health problem that requires intersectoral interventions. Results also showed that a high proportion of children were referred from other hospitals with insufficient resources and training, suggesting an opportunity for intervention in prehospital care in both the initial approach of the injured child and their transfer from the scene to the appropriate hospital for continued health care. Most studies on paediatric trauma in this region have been descriptive. Our study is the first in Colombia and Latin America to assess survival prediction in children with trauma using the PTS. Although international authorities have historically recommended the use of PTS in the injured, it is not routinely used in our healthcare settings; consequently, children are not transferred from trauma sites with objective criteria such as severity classification. Rather, criteria upon transfer are subjective or administrative, delaying the definitive care of more seriously injured patients.

**Implications of all the available evidence**

Our results showing that high levels of violence are the leading cause of death among adolescents will facilitate the proposal of multidisciplinary and intersectoral intervention strategies of a preventive nature. The findings support the systematic use of PTS in the care of injured children, thereby optimising their transfer to the appropriate hospital. This prevents wasting of the resources of large centres in the care of trivial injuries or delaying the care of seriously injured children in centres with insufficient resources, directly affecting patient outcomes.

Alt-text: Unlabelled box

## Introduction

According to the Global Burden of Disease Study, the health of the population worldwide has improved; however, this improvement has been modest in children older than 10 years, in whom injuries continue to be the leading cause of death and disability.^1^^,^^2^ Injuries cause more years of potential life to be lost before 18 years than sudden infant death, cancer and infectious diseases combined.^3^ Unintentional injuries are the second leading cause of death after 5 years of age, and intentional injuries are the third leading cause after 15 years.^1^ In Colombia, violence is the leading cause of death after 5 years of age.^4^ These high injury rates represent a severe public health problem requiring multisectoral collaborative efforts, and are an evident opportunity for preventative injury intervention. However, when prevention fails, the next opportunity for intervention is to provide optimal care for the injured to impact their survival favourably with the fewest sequelae possible. Strategies such as using trauma scores have been proposed; such scores have existed for more than 40 years and have shown variable performance results. Nonetheless, each scoring system has particular benefits that must be considered, along with the ease of application and availability of the resources necessary to calculate the scores.^5^ Trauma scores have prognostic utility, standardise the language used by different professionals (prehospital healthcare workers, emergency physicians, intensivists, surgeons, traumatologists, epidemiologists, and researchers), allow interhospital comparisons, and improve resource planning by providing information about patient severity. The Pediatric Trauma Score (PTS), designed for children, is an available trauma scoring tool. The American College of Surgeons Committee on Trauma (ACS COT) in Advanced Trauma Life Support has highlighted the PTS as a helpful tool for the initial approach to paediatric trauma.^6^ Its performance has not been assessed in Colombia, a region with trauma characteristics and care systems that differ from the United States, where the score was developed. The present study is the first in Colombia and Latin America to evaluate the ability of the PTS to predict survival in injured children. This study also aimed to describe the trauma characteristics and outcomes of the assessed children.

## Methods

### Study design and setting

This was an analytical observational study of a retrospective cohort of children admitted between January 2011 and May 2019 to the emergency room of Fundación Valle del Lili University Hospital in Cali, Colombia. Initially, the study period was proposed until December 2018; however, the period was extended until May 2019 to achieve an optimal sample size.

The study hospital has 523 beds; 205 are in the intensive care unit. The hospital treats highly complex pathologies, including severe trauma, and is equivalent to a major trauma centre. Colombia is a country in northern South America, with 48,258,494 inhabitants.^7^ According to the World Bank classification, the country is categorised as a medium-high income country.^8^ Cali is a city in the southwestern region with 2,496,442 inhabitants.^9^

The study protocol was approved by the hospital's Biomedical Research Ethics Committee under registration number 295–2018.

### Participants

**Figure 1:**
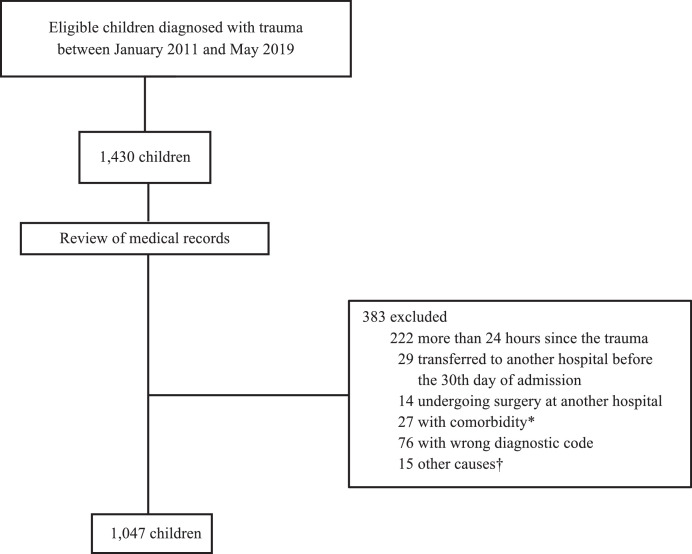
**Participant flow chart**. *Haematological, oncological, metabolic, or osteopathic comorbidities. †Other causes: unknown time since trauma, escape, transfer from provinces other than Valle del Cauca or Cauca.

Participants included children under 18 years of age who presented to the emergency room with a trauma diagnosis (International Classification of Diseases, 10th edition, discharge codes between S00 and T149) and required hospitalisation consisting of a stay longer than 6 h or death before that time. Patients were excluded if they had burns, were involved in a drowning accident, were diagnosed as having a foreign body, or were intoxicated. Further exclusions included if more than 24 h had elapsed since the trauma, if they were transferred to another hospital before day 30 of hospitalisation due to the impossibility of verifying their vital status at that time, or if they were transferred from provinces other than Valle del Cauca or Cauca. In addition, patients were excluded if they had haematological, oncological, metabolic, or osteopathic comorbidities that could influence the approach or outcome, or if they had undergone surgical interventions at another institution due to the impossibility of classifying their initial lesions; therefore, trauma scores could not be calculated which could lead to an erroneous classification of individuals (Figure 1).

### Data sources and measurement

The clinical registries of the included patients were reviewed; diagnostic coding was verified, sociodemographic information, the mechanism and cause of trauma, and the type and severity of injuries were obtained, and radiology reports were reviewed. Trauma time was defined as the time elapsed between injury and hospital admission. This information was collected from the patient's clinical history which included the date and time of the injury. Clinical variables such as heart rate, respiratory rate, blood pressure, Glasgow Coma Scale score, and the variables necessary to calculate the PTS, among others, were obtained. The primary outcome variable was mortality at discharge, measured on day 30 of hospitalisation or earlier if the patient died or was discharged home. We considered surgery, blood transfusion, and intensive care as secondary outcome variables. The predictor variable was the PTS. The injury severity score (ISS), a score widely used by trauma researchers, was calculated and compared between living and dead patients and included in the analysis.^10^

The direct cause of death was determined from death certificates and verified by reviewing the patients’ clinical registries.

### Pediatric trauma score

The PTS was described in 1987 by Tepas et al.^11^ Its application in children combines physiological and anatomical variables—weight, airway status, systolic blood pressure, central nervous system status, fractures, and wounds—to classify the severity of the injury. The tool yields a score between -6 and +12, with lower scores indicating greater severity. A score ≤8 suggests that the patient should be transferred to a trauma centre because of a significantly increased risk of mortality.^12^

### Injury severity score

The ISS was first described in 1974 as the sum of the squares of the three highest Abbreviated Injury Scale (AIS) scores between different body areas.^13^ A score of 16 or more has traditionally been established as a severe injury.^10^ The AIS, necessary to calculate the ISS, was described in 1971, and its score ranges from 0 to 6, with 3 being a severe injury and 6 being the worst injury.^14^

### Statistical methods

Categorical variables are presented as frequencies and proportions and were compared between survivors and deceased using either a chi-square or Fisher's exact test, as appropriate. Continuous variables are presented as the mean and standard deviation or median and interquartile range (IQR) according to the normality of their distribution. Variables were compared between the deceased and survivors using the Student's t-test or Wilcoxon-Mann-Whitney sum of ranges, as appropriate. The performance of PTS in predicting survival was evaluated using a simple logistic regression model, considering PTS as a predictor variable and vital status at discharge (alive or dead) as a dichotomous outcome variable, with the application of statistical tools such as discrimination and calibration. Discrimination using the area under the receiver-operating characteristic (AUROC) curve accurately demonstrates the ability of the score to predict survival, with a value of 1 indicating perfect discrimination and a value of 0·5, a predictive capacity that is not superior to chance. Calibration was assessed using the Hosmer-Lemeshow goodness-of-fit test, which measures the agreement between the observed probability of survival and that predicted by the model, indicating *p* > 0·05 as a good calibration.^15^

We hypothesised a good discriminative capacity with an ROC curve greater than 0·9 and a good fit between observed and expected mortality with a goodness of fit of *p* > 0·05. The sample size was calculated using a proportional-comparison formula. The a priori estimated sample size was 1043 children, which was estimated to detect a difference of 2% between observed and expected mortality (two-sided 5% significance, power 90%).

The analyses were performed using Stata® version 16.1 (StataCorp, College Station, Texas 77845, USA).

### Role of the funding source

There was no funding source for this study. All authors had full access to the study data and final responsibility for the decision to submit for publication.

## Results

Between January 2011 and May 2019, 1047 children aged <18 years were admitted to the emergency room and fit the criteria to be included in the study. Among those excluded, 29 (2·7%) were transferred to hospitals of a lower level of complexity before the 30th day of hospitalisation. These patients were excluded because their vital status could not be verified at discharge. Reasons for transferring a patient before 30 days of hospitalisation were related to administrative issues affecting the completion of treatment. Thus, 29 patients, considered a low proportion, were transferred with no 30-day survival data. However, these patients likely were alive and thus would have a minimal effect on survival estimates.

**Figure 2:**
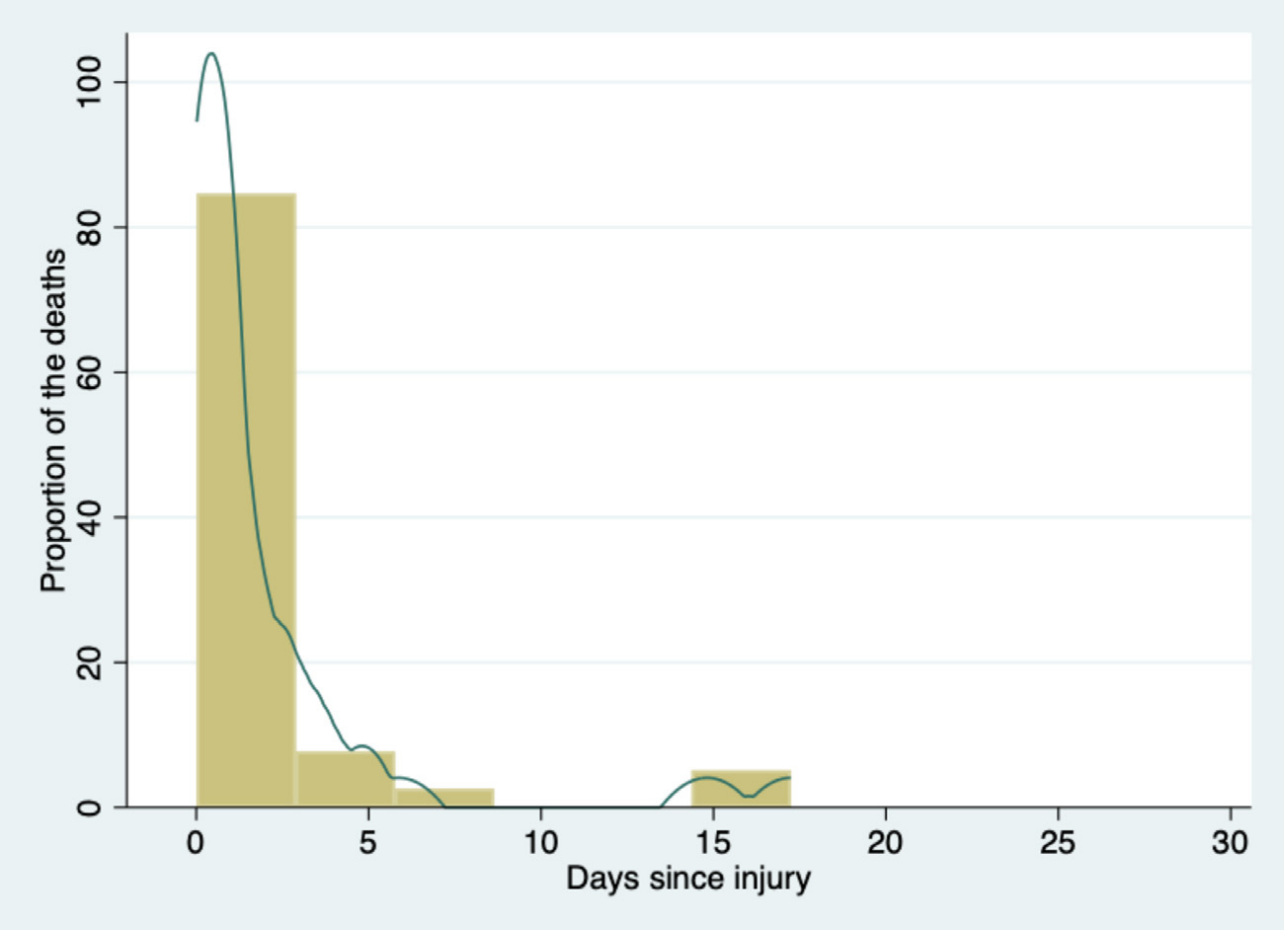
**Mortality timing**. Mortality was measured on day 30 of hospitalisation or earlier if the patient died or was discharged home. Data were available for 815 patients.

The median age was 12 years (IQR, 5–15 years), and 73·7% were male. Colombia's subsidised health insurance system covered 34% of the total, and the remaining 66% was covered by the contributory health insurance system (private insurance). The injury mechanisms were blunt (66·1%) and penetrating (33·9%). The most common causes of injury were traffic accidents and assaults, accounting for 31·5% and 29%, respectively; the former was more frequent in children under 10 years of age, and the latter was more frequent after 10 years of age. The median PTS was 7 (IQR, 5–9). The mortality rate was 5·9% (Table 1); mortality occurred in the first 24 h in 67% of cases, and the median was 15 h (IQR 3·8–49) (Figure 2) without deaths after day 20 of hospitalisation. Cranioencephalic trauma was the most frequent cause of death (Table 2). Sixty-one and three tenths percent of the deaths were due to intentional trauma, with firearms caused 95% of these deaths. Table 2 shows the cause of death according to the anatomical location and intentionality of injury.

**Table 1:** Sociodemographic and clinical characteristics of the children.

	*n* = 1047
Median age, years (p25 to p75)	12 (5–15)
Age groups	
0–4 years	221 (21·1%)
5–9 years	229 (21·9%)
10–14 years	249 (23·8%)
15–17 years	348 (33·2%)
Sex	
Male	772 (73·7%)
Female	275 (26·3%)
Payor status	
Private insurance	692 (66%)
Subsidised	355 (34%)
Origin (province)	
Valle del Cauca	775 (74%)
Cauca	272 (26%)
Transportation^a^	
Ambulance	791 (76·1%)
Private/not ambulance	248 (23·9%)
Transferred from another hospital^b^	
Yes	725 (70%)
No	311 (30%)
Mechanism of injury	
Blunt	692 (66·1%)
Penetrating	355 (33·9%)
Cause of injury	
Traffic accident	330 (31·5%)
Assault	304 (29%)
Firearm	224
Stabbing	59
Hit by/against something	13
War explosive	8
Fall	297 (28·4%)
Other	110 (10·5%)
Unknown	6 (0·6%)
Age-adjusted hypotension	
Yes	74 (7%)
No	973 (93%)
Glasgow Coma Scale^c^	
13–15	790 (78%)
9–12	79 (7·8%)
3–8	144 (14·2%)
PTS, median (p25 to p75)	7 (5–9)
ISS, median (p25 to p75)	9 (5–17)
ICU	
Yes	521 (49·8%)
No	526 (50·2%)
Blood transfusion	
Yes	265 (25·3%)
No	782 (74·7%)
Surgery	
Yes	649 (62%)
No	398 (38%)
Hospital stay, median (p25 to p75)	3·5 (1·7–6·9)
Mortality	62 (5·9%)

Data are *n* (%) or median (p25 to p75). PTS=pediatric trauma score. ISS=injury severity score. ICU=intensive care unit.

a Data were available for 1039 patients.

b Data were available for 1036 patients.

c Data were available for 1013 patients.

**Table 2:** Cause of death according to intentionality and anatomical site of the injury.

	Head	Chest	Abdomen	Thoracoabdominal
Unintentional	20	1	1	1
Intentional	32	1	2	3
Indeterminate	1			

Data are *n*.

Regarding the secondary outcome variables, 62% underwent surgery, 49·8% were admitted to the ICU, and 25·3% received a blood transfusion.

For bivariate analysis, the patients were classified according to their vital status at discharge (Table 3).

**Table 3:** Bivariate analysis: characteristics of patients by vital status at discharge.

	Alive (*n* = 985)	Dead (*n* = 62)	*p* value
Median age, years (p25 to p75)	11 (5–15)	15 (13–17)	<0·0001
Age groups			<0·0001
0–4 years	215 (21·8%)	6 (9·7%)	
5–9 years	225 (22·8%)	4 (6·4%)	
10–14 years	235 (23·9%)	14 (22·6%)	
15–17 years	310 (31·5%)	38 (61·3%)	
Sex			0.03
Male	719 (73%)	53 (85·5%)	
Female	266 (27%)	9 (14·5%)	
Payor status			<0·0001
Private insurance	667 (67·7%)	25 (40·3%)	
Subsidised	318 (32·3%)	37 (59·7%)	
Origin			0·24
Valle	733 (74·4%)	42 (67·7%)	
Cauca	252 (25·6%)	20 (32·3%)	
Transferred from another hospital^a^	672 (69%)	53 (87%)	
Mechanism of injury			<0·0001
Blunt	667 (67·7%)	25 (40·3%)	
Penetrating	318 (32·3%)	37 (59·7%)	
Age-adjusted hypotension	59 (6%)	15 (24·2%)	<0·0001
Glasgow Coma Scale, median (p25 to p75)^b^	15 (14–15)	3 (3–6)	<0·0001
PTS, median (p25 to p75)	8 (5–9)	0 (0–1)	<0·0001
ISS, median (p25 to p75)	9 (4–16)	31 (25–37)	<0·0001
ICU			<0·0001
Yes	470 (47·7%)	51 (82·3%)	
No	515 (52·3%)	11 (17·7%)	
Blood transfusion			<0·0001
Yes	232 (23·6%)	33 (53·2%)	
No	753 (76·4%)	29 (46·8%)	0·08
Surgery			
Yes	617 (62·6%)	32 (51·6%)	
No	368 (37·4%)	30 (48·4%)	<0·0001

Data are *n* (%) or median (p25 to p75). PTS=pediatric trauma score. ISS=injury severity score. ICU=intensive care unit.

a Data were available for 1036 patients.

b Data were available for 1013 patients.

Eighty-four percent of the deceased patients were between 10 and 17 years of age, and 71% of them died due to an assault. Most deceased patients were adolescent males with a lower ability to pay, penetrating trauma, admitted in a coma (Glasgow median, 3), with a median PTS of 0, and a median ISS of 31 (severe trauma). Almost 25% were admitted to the hospital in a state of decompensated shock (hypotension adjusted for age), indicating a state of extreme severity, with death occurring within the first hour after admission (median, 60 min; IQR, 45–94 min). In this group of dead children, 51·6% underwent surgery, 53·2% received a blood transfusion, and 82·3% were admitted to the ICU since the remaining percentage died before ICU admission.

We found 689 (66%) patients with a PTS ≤8 and 358 with a PTS >8. The performance of the PTS showed an AUROC of 0·93 (95% CI 0·89 to 0·96; *p* < 0·0001) (Table 4; appendix p 2) and Hosmer–Lemeshow goodness of fit of 7·97 (*p* = 0·33; Table 4), indicating excellent discrimination and good calibration, respectively, and a performance that was maintained for the different injury mechanisms (blunt or penetrating) (Table 4). Given the classic PTS cut-off point of 8 as the value from which mortality increases significantly, we evaluated the performance of the PTS by categorising its score, observing an AUROC of 0·92 (95% CI 0·89–0·94) in those with PTS ≤8 and 0·74 (95% CI 0·71–0·76) in those with a PTS >8. We “rescued” the predictive capacity in children with a worse score for whom referral to the appropriate hospital is a priority because of their high probability of dying. An additional analysis was done to evaluate the performance of the PTS according to age and the presence of severe head injury (Appendix p 3).

**Table 4:** Performance of Pediatric Trauma Score by the mechanism of injury.

	OR	*p* value	95% CI	AUROC	HL	Prob > X2
PTS						
Complete cohort (*n* = 1047)	1·8	<0·0001	1·61–2·02	0·93	7·97	0·33
Blunt trauma (*n* = 692)	1·68	<0·0001	1·44–1·95	0·91	3·19	0·87
Penetrating trauma (*n* = 355)	1·9	<0·0001	1·59–2·28	0·93	8·72	0·12

OR=odds ratio, CI=confidence interval, AUROC=area under the receiver-operating characteristic curve, HL=Hosmer–Lemeshow.

## Discussion

This study presents data on the frequency, severity, and characteristics of trauma in a paediatric sample in Colombia. Traffic accidents and assaults were the most frequent causes of injury. The results regarding traffic accidents are in accordance with existing evidence from other researchers worldwide^16^, ^17^, ^18^, ^19^, ^20^ and within the region.^21^^,^^22^ These injuries are potentially preventable with the implementation or optimisation of adequate, exhaustive, and strong prevention measures linked to a solid and robust surveillance system. Notably, reported deaths in our study were predominantly intentional and caused by firearms, which contrasts with studies reporting that deaths due to trauma were mostly unintentional.^1^ We also observed that most of the deceased children had severe cranioencephalic trauma and that death occurred quickly, similar to studies that analysed trauma mortality.^23^ The proportion of deceased who did not receive surgery or require admission to the ICU was attributed to the extreme severity of their admission status, with rapid progression to brain death and death. Although mortality from injuries has decreased worldwide, injuries remain among the leading causes of death,^1^ and many young lives continue to be lost. In Colombia, violence is the leading cause of death after 5 years of age^4^; this is a serious and critical social problem and of major concern considering that these victims are children. These deaths are “the tip of the iceberg” as more injured children survive but require hospital care or are left with disabilities.^16^ The considerable percentage of hospitalised patients who were transferred from hospitals with a lower level of complexity, mainly those who died, is a noteworthy finding of our study. We do not have information on the treatment received in the originating hospitals; however, the patient status upon arrival included coma and median PTS and ISS scores of 0 and 31, respectively, values with a high mortality rate.^12^^,^^24^ Furthermore, a proportion of them arrived hypotensive, which in children is considered a terminal situation with increased mortality^25^; therefore, it is crucial to receive the required care in the shortest time possible. These critical patients should be transferred directly from the injury site to a major trauma centre because the risk of imminent death is high without adequate care and timely intervention.^26^^,^^27^

The proportion of violent trauma evidenced in this study represents a serious public health problem that must be addressed with investment and combined efforts at the local, international and intersectoral levels.^28^ Such efforts include those proposed by the World Health Organization (WHO) in collaboration with the United States Centers for Disease Control and Prevention (US CDC), the Pan American Health Organization (PAHO), and the United Nations Children's Fund (UNICEF), among other organisations, with the implementation of the INSPIRE strategies for preventing violence against children.^29^ Although the prevention of injuries is an ideal concept, injuries do occur despite the best preventive efforts, and physicians must be prepared to treat them accordingly. One strategy with demonstrated value is the use of trauma scores^30^ that objectively classify patients and facilitate routing to an appropriate trauma centre; such scores have been used in paediatrics since the 1980s. However, their performance in survival prediction has not been evaluated in Latin America. The PTS was designed in a developed country (United States), with social characteristics different from those of our country, as well as an organised road infrastructure, a more specialised, structured and coordinated health and trauma care system, and trauma characteristics different from those of our environment. Additionally, the PTS has been implemented for several decades, during which time there have been progressive technological developments and the creation of particular protocols for caring for injured patients. It was important to assess the effectiveness of the PTS in our environment. We found that the PTS was considerably lower in deceased patients. The critical value at which infant mortality has traditionally been considered to increase was reported by Tepas as 8.^12^ However, in our study, we evaluated the cut-off point in the ROC curve with greater sensitivity and specificity, finding this threshold at 4, very different than the threshold proposed by Tepas.^12^ A study by Orliaguet et al.^31^ had similar results to ours. Like our study, their study used ROC curves, one of the statistical tools with greater diagnostic accuracy in this type of analysis.^32^^,^^33^ We evaluated the capacity of the scoring tool to predict survival, finding an excellent performance in both discrimination and calibration, which has also been demonstrated by researchers in other countries.^17^^,^^31^^,^^34^, ^35^, ^36^ Other studies, such as that of Yoon et al.^37^ reported that although the performance of the PTS was good, it was lower than that of other scores such as the BIG score (composed of the base deficit [B], International normalized ratio [I], Glasgow Coma Scale [G]). However, those scores require laboratory tests, resulting in additional time waiting for results or a complex calculation in some contexts. Furthermore, it may not be available at all levels of care or in the prehospital setting. There are several reasons for the good performance of the PTS found in our study. First, PTS combines anatomical and physiological factors, including weight, among its variables, recognising that the severity of injuries and responses differ depending on age or body size.^5^^,^^38^ Second, PTS evaluates the central nervous system, which is of great relevance because with multisystem involvement as often occurs with severe trauma, the level of consciousness is one of the main signs of hypoperfusion and haemodynamic instability that occurs even before hypotension. Third, as an element of quality control, the PTS makes it possible to identify deaths with less severe injuries, in which handling errors could have occurred. However, the current study did not address the characteristics of the treatment received as this was not part of the stated objectives. Added to these strengths is the ease of calculation of the PTS in both prehospital and in-hospital settings by medical or non-medical health personnel. In addition to its good results, it is reasonable to systematically include PTS in the care for injured children, as the American College of Surgeons recommends in its Advanced Trauma Life Support (ATLS) manual.^6^ The PTS quantifies the severity of trauma, supports efficient resource allocation and use for the care of injured children and the dissemination of data and experiences in hospitals, and generates scientific evidence that objectively helps in public health decision-making. With its use, we, as healthcare providers in our country, could contribute to and align ourselves with the Global Strategy for the Health of Women, Children, and Adolescents, which seeks, among other objectives, to reduce preventable mortality and improve health for children and adolescents by 2030.^39^

This study had the following limitations. The study was retrospective with inherent limitations. We did not have information about prehospital care, the treatment received before arrival at our hospital, or the specific circumstances under which the trauma occurred, such as the use of safety measures in traffic accidents. The study was monocentric, which limits the generalisability of the results. Including other geographical contexts implies collaborative inter-institutional efforts, but gives us privileged information, which will be interesting to consider in the future. Regarding the strengths of our study, the sample size was appropriate compared to other studies on paediatric trauma in the region and included a significant proportion of patients with violent trauma, which is low in most paediatric studies.

In conclusion, most deaths in this study were caused by intentional trauma (violence), and firearms were used in almost all deaths. Cranioencephalic trauma was the leading cause of death. PTS showed an excellent ability to predict survival, regardless of the mechanism of injury. Prospective studies are needed; ideally, these would include the analysis of regional trauma records to allow epidemiological surveillance at the hospital level. Such studies would complement the surveillance carried out by government entities, and thus reduce the knowledge gap and enhance planning strategies that would impact the health and well-being of children.

## Contributors

ADlR-P, AG and AF-L contributed to the conceptualisation, methodology, statistical analysis, and interpretation of data and administered the project. ADlR-P did the data curation and wrote the original draft. AG and AF-L provided academic resources, supervised the project, and validated the data. LC and SR contributed to data collection and wrote different draft versions. All authors had full access to all of the data, critically reviewed and approved the manuscript's final version, and had final responsibility for the decision to submit for publication.

## Data sharing statement

According to the Helsinki Declaration, the datasets generated and analysed during the current study are not publicly available to protect the rights of research participants. However, these are available from the corresponding author upon reasonable request.

## Declaration of interests

We declare no competing interests.

## Appendix Supplementary materials

Image, application 1
