# Supplementary material for: Performance of the Paediatric Trauma Score on survival prediction of injured children at a major trauma centre: A retrospective Colombian cohort, 2011–2019
Source: Lancet Reg Health Am. 2022 Jun 29;13:100312. doi: 10.1016/j.lana.2022.100312 (PMC9903890; doi:10.1016/j.lana.2022.100312)
Supplement: Supplementary file 1 [file mmc1.docx]

**Table of Contents**

Figure 1: Performance of Pediatric Trauma Score on survival prediction …………….…….2

Table 1: Performance of Pediatric Trauma Score by age group and severe head trauma….….3

**Figure 1. Performance of Pediatric Trauma Score on survival prediction**

The performance was evaluated using discrimination. Area under ROC curve shows high accuracy in predicting survival. ROC=receiver-operating characteristic.

|  | **OR** | **p value** | **95% CI** | **AUROC** | **HL** | **Prob > X2** |
| --- | --- | --- | --- | --- | --- | --- |
| Age group (dichotomized) |  |  |  |  |  |  |
| <15 years (n=699, dead n=24) | 0·51 | <0·0001 | 0·36–0·65 | 0·94 | 3·53 | 0·83 |
| 15–17 years (n=348, dead n=38) | 0·66 | <0·0001 | 0·47–0·84 | 0·91 | 27·7 | 0·00 |
| Severe head injury (AIS ≥3) |  |  |  |  |  |  |
| Yes (n=369, dead n=54) | 0·52 | <0·0001 | 0·38–0·67 | 0·89 | 4·32 | 0·74 |
| No (n=678, dead n=8) | 0·54 | <0·0001 | 0·31–0·78 | 0·82 | 11·27 | 0·05 |
| OR=odds ratio, CI=confidence interval, AUROC=area under the receiver-operating characteristic curve, HL=Hosmer–Lemeshow, AIS= Abbreviated Injury Scale | | | | | | |
| **Table 1. Performance of Pediatric Trauma Score by age group and severe head trauma** | | | | | | |
